# Influence of Iron on Cytotoxicity and Gene Expression Profiles Induced by Arsenic in HepG2 Cells

**DOI:** 10.3390/ijerph16224484

**Published:** 2019-11-14

**Authors:** Yonghua Wang, Yuxuan Liu, Su Liu, Bing Wu

**Affiliations:** 1 Key Laboratory of Integrated Regulation and Resource Development on Shallow Lakes of Ministry of Education, College of Environment, Hohai University, Nanjing 210098, China; l555b6@126.com; 2 State Key Laboratory of Pollution Control and Resource Reuse, School of the Environment, Nanjing University, Nanjing 210023, China; su_19881101@126.com (S.L.); bwu@nju.edu.cn (B.W.)

**Keywords:** arsenic, iron, gene expression profiles, cytotoxicity, oxidative stress

## Abstract

The toxicity of arsenic (As) could be influenced by many environmental factors and elements. Iron (Fe) is one of the elements that could be involved in As-induced toxicity. In this study, the interactive effects of Fe and As in HepG2 cells were analyzed based on cytotoxicity and transcriptomic analyses. The results showed that Fe could decrease cell viability and increase mitochondrial depolarization induced by As exposure. Oxidative stress and damage have been proven to be one of the main mechanisms of As toxicity. Our results showed that Fe increased the generation of reactive oxygen species (ROS) and lipid peroxidation product malondialdehyde (MDA) induced by As exposure. Microarray analysis further verified that Fe increased the alteration of gene expression and biological processes related to oxidative stress, cell proliferation, and the apoptotic signaling pathway caused by As exposure. Both results of cytotoxicity and transcriptomic analyses suggest that an increase of Fe in the human body could increase the As-induced toxicity, which should be considered during the health risk assessment of As.

## 1. Introduction

Arsenic (As) exposure is a worldwide public health concern [1]. Epidemiological studies and clinical observations indicate that As exposure is associated with an increased incidence of human cancer of the skin, liver, kidney, lung, prostate, and urinary bladder [2,3,4]. Besides the carcinogenic effect, epidemiological studies in highly exposed populations have also revealed strong links between arsenic exposure and skin lesions, hypertension, cardiovascular disease, diabetes, and respiratory disease [5,6,7,8]. Recently, the influence of other elements on As-toxicity has received more and more attention, which could change the real risk of As exposure in the environment. For example, vitamins, fruit, tea, glutathione, N-acetylcysteine, and zinc could reduce As-induced toxicity by increasing antioxidative enzymes to antagonize oxidative stress caused by As and/or increasing As methylation [9,10,11,12,13]. Similar interactive effects could also be found in other metals [14]. 

Iron (Fe) is a common element in the environment and human biological system, which is generally considered to have no toxic effects on humans. However, some researchers have found that Fe could be involved in As-induced oxidative damage and toxicity [15,16]. Oxidative stress has been proposed as a main mechanism of As toxicity [17,18]. The As species in the human body can cause Fe release from ferritin, which plays an important role in the storage of free Fe [19]. Endogenous free Fe participates in generating harmful oxygen species by Fenton reaction, which can induce oxidative stress and damage. Thus, the combined effects of Fe and As have received attention. When exogenous Fe and As were co-exposed by drinking water, earlier evidence showed that ferrous sulfate could reduce the clastogenic effects of As in mice [20], whereas a recent study revealed different findings—that there was a synergistic interaction between As_2_O_3_ and Fe in relation to rat livers [21]. Our previous studies found that Fe in drinking water could decrease As toxicity in mice by the co-flocculation of As and Fe in gastrointestinal tract [22]. However, we also found that the co-exposure of As and Fe induced synergistic effects in the cultural cell line. These results imply that the endogenous Fe in body (tissue) and exogenous Fe in drinking water (and food) could induce different effects on As toxicity. However, mechanisms of the synergistic effect of As and Fe are unclear, which could provide insight for the identification of their interactive effects and human health risk.

In this study, we determined the combined cytotoxicity of As and Fe and their underlying mechanisms in HepG2 cells. Influences of Fe on inorganic As-induced decrease in cell viability, reactive oxygen species (ROS) generation, mitochondrial depolarization, and oxidative damage were investigated by treating with As and Fe alone or in combination for 24 h. The gene expression profiles in HepG2 cells were characterized by cDNA microarray analysis. Combined with the above phenotypic toxicity, bioinformatics analysis was applied to determine the biological meaning of differentially expressed genes to characterize potential mechanisms of the synergistic effects of Fe and As.

## 2. Materials and Methods

### 2.1. Cell Culture and Treatment

Human hepatoma cell line HepG2 obtained from KeyGEN Biotech (China) was maintained in high glucose Dulbecco’s modified eagles medium (DMEM) with 10% sterile fetal bovine serum (FBS) under standard cell culture conditions (37 °C, and 5% CO_2_). Before being treated with As and Fe, HepG2 cells (10–18 passage) at a density of 10,000 cells per well were allowed to attach in 96-well plates for 24 h. Then, the HepG2 cells were exposed to As and/or Fe in DMEM medium for 24 h, and were used for further analyses. The exposure concentrations of As and Fe were set to 20 μM and 100 μM, respectively, which were based on their actual blood or urinary concentrations and reported effect concentrations [22,23]. Arsenic oxide was obtained from NSI Solution Inc. (Raleigh, NC, USA). Ferric chloride was obtained from Sigma Chemical Co. (St.Louis, MO, USA).

### 2.2. Cytotoxicity Test

Cell viability was determined by CCK-8 (Dojindo Molecular Technologies, Inc. Kumamoto, Japan). After As and/or Fe exposure, 10 μL of CCK-8 solution was added into each well of the 96-well plate. Then, the plate was incubated for 2 h at 37 °C. Spectrophotometric measurements were performed in a microplate reader (Synergy H1, BioTek, Winooski, VT, USA) at a wavelength of 450 nm. Based on the absorbance, the cell viability in the treated group was expressed as the percentage of viable cells compared with that of untreated control cells.

### 2.3. Mitochondrial Membrane Potential Assay

Mitochondrial membrane potential was analyzed by JC1 kit (KeyGEN, Nanjing, China). The JC1 is a lipophilic cationic probe which can enter into the mitochondria, where it accumulates and starts forming reversible complexes called J aggregates. The J aggregates exhibit excitation and emission in the red spectrum, which is different from the green spectrum of the JC1 molecule. In this study, after exposure, 50 μL JC1 solution (final concentration was 20 nM) was added into each well of the 96-well plate. The plate was incubated for 25 min at 37 °C. Then cells were washed twice with PBS buffer and analyzed on a microplate reader (Synergy H1, BioTek, Winooski, VT, USA) at two groups of fluorescence: green (excitation wavelength: 485 nm and emission wavelength: 530 nm) and red (excitation wavelength: 530 nm and emission wavelength: 590 nm). Mitochondrial membrane potential was determined by the ratio of red to green fluorescence intensity. A decrease in the ratio indicated mitochondrial depolarization, which participates in the induction of the apoptotic signaling pathway.

### 2.4. Oxidative Stress Analysis

Intracellular ROS were measured by DCFH-DA (molecular probes). DCFH-DA can passively enter the cell and hydrolyze to form DCFH, which is a nonfluorescent molecule and can be oxidized to fluorescent dichlorofluorescein (DCF) by cellular oxidants. During DCF assay, Hoechst 33342 (YeaSen Bio-technology, Shanghai, China) was used as a proxy measurement for the number of HepG2 remaining in each well. After Fe and/or As exposure, 50 μL DCF (final concentration 10μM) was added into each well of 96-well plate. After incubating for 25 min at 37 °C, the cells were washed twice with PBS. Then, 100 μL Hoechst 33342 (5 μg/ml) was added into each well. After incubating for 15 min at 37 °C, the cells were washed by PBS again and measured using a microplate reader (Synergy H1, BioTek, Winooski, VT, USA). The excitation and emission wavelength for DCF were 485 nm and 530 nm, respectively. The excitation and emission wavelengths for Hoechst 33342 were 350 nm and 460 nm, respectively. The fluorescence values of DCF were normalized by Hoechst fluorescence values in the same well to avoid test error due to the loss of cell numbers during the exposure experiment.

The oxidative stress and damage of cells was further determined by the measurement of superoxide dismutase (SOD) and lipid peroxidation product malondialdehyde (MDA). A total of 10^7^ cells were collected by trypsinization, followed by homogenization and centrifugation at 3000 rpm for 5 min, and the supernatant was used for various estimations. The SOD activity and MDA level were measured using commercial kits (Jiancheng, Nanjing, China), which were determined by the absorbance of the microplate reader at a wavelength of 450nm and 532 nm, respectively. Then, the SOD activity and MDA level were expressed as U/mg protein and nmol/mg protein, respectively. The protein concentration was determined by using the Coomassie Brilliant Blue method (Jiancheng, Nanjing, China). Each experiment was performed in triplicate.

### 2.5. Microarray Analysis

Gene expression analysis was conducted by the cDNA Microarray. Total RNA extraction was performed using the Takara RNA Kit (Takara Biotechnology, Tokyo, Japan) and the quantity of total RNA was determined by a NanoDrop 2000. The cDNA synthesis was carried out in a 10 μL reaction solution containing 500 ng RNA and reverse transcription mix (Invitrogen, Waltham, MA, USA). The synthesized cDNA generated from the total RNA was fragmented and labeled using the Affymetrix GeneChip® WT Terminal Labeling kit, then hybridized to an Affymetrix GeneChip PrimeView Array (Affymetrix, Santa Clara, CA, USA) and scanned using an Affymetrix GeneChip Scanner 7G [24]. Microarray data quality analysis was performed using Affymetrix Expression Console software. Probe sets labeled as control were excluded from further analysis. The associated *p*-values for moderated t-statistics were adjusted using the false discovery rate (FDR) method due to multiple hypotheses testing. Differentially expressed genes (DEGs) were analyzed by function and pathway analysis using DAVID 6.7 (http://david.abcc.ncifcrf.gov/tools.jsp) to interpret their biological meaning. Altered biological pathways and processes were identified by mapping DEGs into the Gene Ontology (GO) database (http://www.geneontology.org) and the Kyoto Encyclopedia of Genes and Genomes (KEGG) pathway database (http://www.genome.ad.jp/kegg/pathway.html). 

### 2.6. Real-Time Quantitative PCR (qPCR)

qPCR was applied to verify the results from cDNA microarray analysis based on the MIQE guidelines. First, cDNA was synthesized from 1 μg of total RNA with the Superscript III Reverse Transcriptase (Invitrogen) using random hexamers. The qPCR reactions were carried out on a Corbett Real-Time PCR Machine with the Platinum Taq DNA Polymerase (Invitrogen). The amount of template was quantified with SYBR Green (Invitrogen). The thermocycling regime was 95 °C for 2 min, followed by 40 cycles of 95 °C for 15 s, 54–55 °C for 30 s, and 72 °C for 30 s. Relative levels of target mRNA normalized to β-actin mRNA were calculated based on the 2^−∆∆Ct^ method. The primer sequences used in this study are given in Table 1.

### 2.7. Statistical Analysis

The data were analyzed using the GraphPad Prism 6.0 software (GraphPad Software, San Diego, CA, USA). Statistical differences were evaluated using the one-way analysis of variance (ANOVA) test, followed by Tukey’s post hoc test. The homogeneity and normality of variance were determined by the Brown–Forsythe test and D’Agostino–Pearson test, respectively. Results were expressed as means ± standard deviation (SD). A *p*-value < 0.05 was considered statistically significant.

## 3. Results and Discussion

### 3.1. Cytotoxicity of As and Fe in HepG2

**Figure 1:**
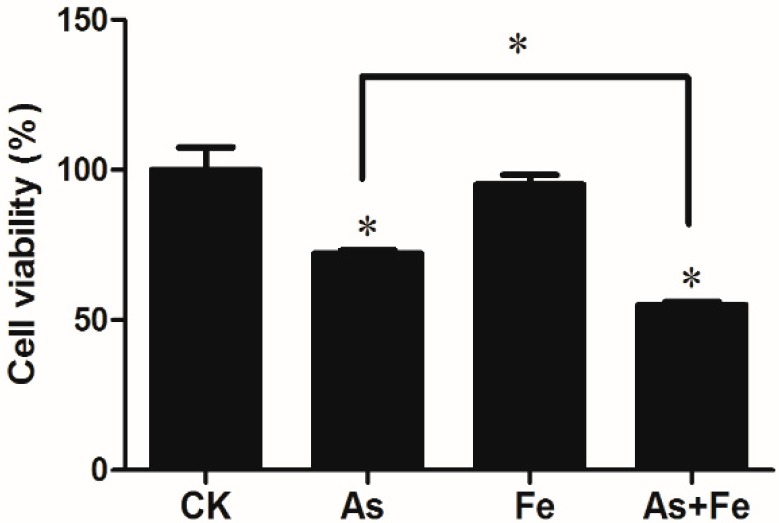
Cell viability of HepG2 exposed to 20 μM As or/and 100 μM Fe. CK, As, Fe, and As + Fe mean the control, arsenic alone exposure, iron alone exposure, and co-exposure of arsenic and iron groups, respectively. The cytotoxicity assays were performed by three independent 96-well experiments. *: *p*-value < 0.05.

Liver is a target organ of arsenic carcinogenesis [25]. Thus, the human hepatoma HepG2 cell line was chosen to evaluate the combined toxicity of As and Fe. Results are shown in Figure 1; 100 μM Fe did not cause a significant decrease in cell viability in HepG2, but the cell viability of HepG2 exposed to 20 μM As was 72%, indicating As induced the cytotoxicity in HepG2 cells. This result is consistent with the previous report on other human lung cells [26]. When HepG2 cells were co-exposed to As and Fe, the cell viabilities were dramatically reduced to 55%, which was significantly lower than the effects of As alone exposure. The results showed the Fe at non-toxicological concentration could significantly increase the As toxicity, indicating the synergistic effects of As and Fe in in vitro cell lines. 

### 3.2. Changes in Mitochondrial Membrane Potential Induced by As and Fe in HepG2 Cell

**Figure 2:**
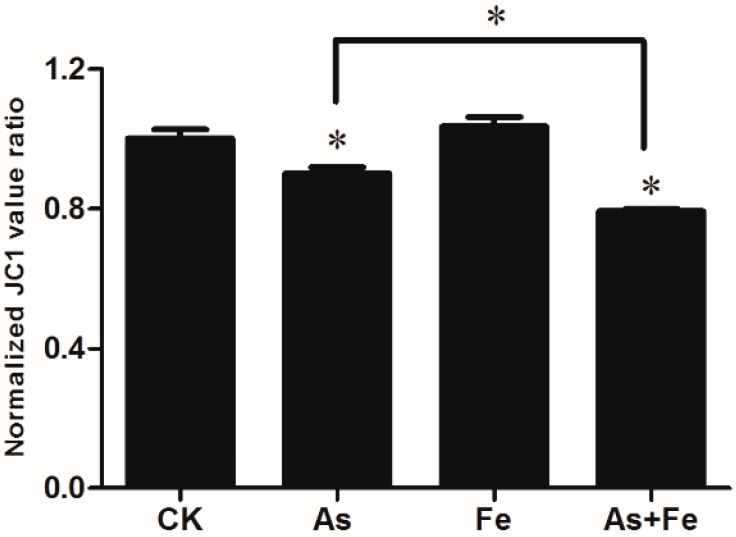
Mitochondrial membrane potential of a HepG2 cell exposed to 20 μM As or/and 100 μM Fe. CK, As, Fe, and As+Fe mean the control, arsenic alone exposure, iron alone exposure, and co-exposure of arsenic and iron groups, respectively. The cytotoxicity assays were performed by three independent 96-well experiments. *: *p*-value < 0.05.

Mitochondrial membrane potential in HepG2 exposed to As and/or Fe was determined by the red/green fluorescence intensity ratio of JC1. The exposure of 20 μM As alone caused a significant decrease in red/green ratios, indicating mitochondrial depolarization (Figure 2). This result is consistent with the reports from previous literature. For example, Kang et al [27] found that after exposure to As_2_O_3_, mitochondrial apoptotic cell death happened in human cervical cancer cells. Jiang et al [28] revealed that As_2_O_3_ inhibits cell growth and induces apoptotic process in gastric cancer cells, involving p53 over-expression and activation of caspase-3. The exposure of 100 μM Fe did not cause changes in mitochondrial membrane potential, but 100 μM Fe could significantly increase the As-induced mitochondrial depolarization, which suggests a synergetic toxic effect.

### 3.3. Determination of Oxidative Stress and Damage in HepG2

**Figure 3:**
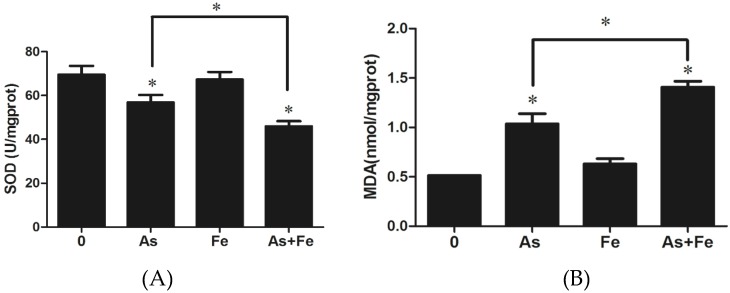

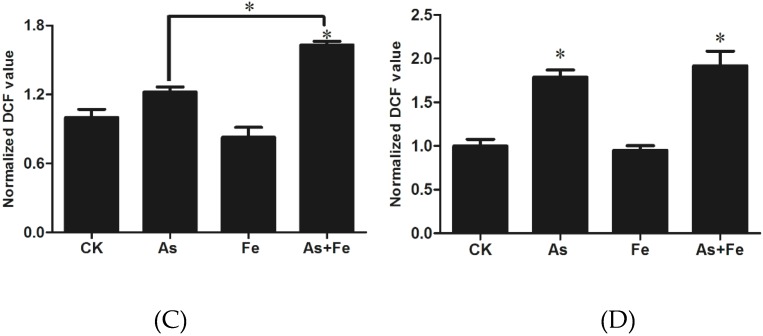
Oxidative stress and damage in HepG2 cells induced by 20 μM As or/and 100 μM Fe. (**A**) and (**B**) show the superoxide dismutase (SOD) activity and malondialdehyde (MDA) levels, respectively. (**C**) shows the normalized dichlorofluorescein (DCF) fluorescence values after exposure to 20 μM As or/and 100 μM Fe, (**D**) shows the normalized DCF fluorescence values after exposure to 2 μM As or/and 50 μM Fe. CK, As, Fe, and As + Fe mean the control, arsenic alone exposure, iron alone exposure, and co-exposure of arsenic and iron groups, respectively. The cytotoxicity assays were performed by three independent 96-well experiments. *: *p*-value < 0.05.

To determine the intracellular oxidative stress and damage, SOD activity and MDA level were measured (Figure 3). Interestingly, the 20 μM As treatment significantly increased MDA level, but a significant decrease in SOD activity was observed under the same treatment. Fe supplementation in the As group significantly increased the production of MDA by 35.8% compared to the As alone group, while SOD activity was further suppressed when 100 μM Fe was added, indicating that Fe might play an important role in the oxidative damage caused by As. Growing evidence has shown that ROS generation associated with As exposure plays a fundamental role in the induction of adverse health effects and disease [17,29,30]. After ROS generation, their effect can be balanced by the antioxidants. One of the most effective intracellular enzymatic antioxidants is SOD. If SOD cannot effectively eliminate the ROS, elevated ROS can result in an attack of cellular components involving polyunsaturated fatty acid residues of phospholipids, and the final product of the peroxidation process is MDA [31]. Our results provide direct evidence that Fe can reduce the activity of antioxidants and increase the oxidative damage caused by As exposure in HepG2 cells. 

In order to validate the above results, we further measured intracellular ROS generation in HepG2 cells. After exposure to 20 μM As, intracellular ROS in HepG2 was significantly increased (Figure 3). When As (20 μM) and Fe (100 μM) were co-exposed to HepG2, compared with the exposure of As alone, no significant difference was found (*p* < 0.05). Since the 20 μM As significantly decreased cell viability, which can influence the measurement of intracellular ROS levels, low As and Fe exposure concentrations were also applied to better characterize their combined effects. When the exposure concentrations of As and Fe were 2 μM and 50 μM, respectively, Fe significantly increased the ROS level induced by As, indicating that the oxidative stress of As was increased by Fe. 

### 3.4. Gene Expression Profiles in HepG2 Exposed to As and Fe

**Figure 4:**
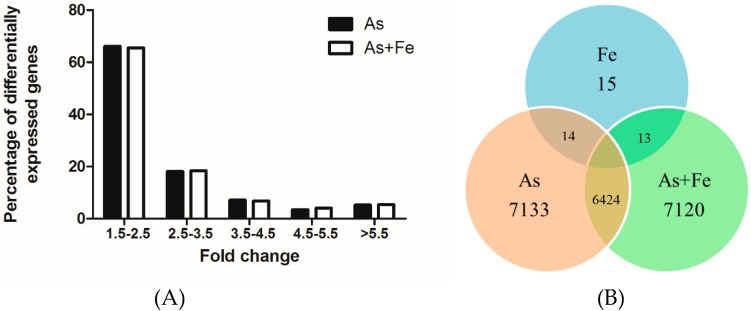
Number of differentially expressed genes in As and As + Fe groups. (**A**) Distribution of differentially expressed genes in different ranges of fold change. (**B**) Venn diagram of differentially expressed genes in treated groups. CK, As, Fe, and As + Fe mean the control, arsenic alone exposure, iron alone exposure, and co-exposure of arsenic and iron groups, respectively.

Gene expression profiles in HepG2 cells exposed to 20 μM As and/or 100 μM Fe were analyzed using cDNA microarray. The criteria were that *p*-values less than 0.05 and fold change higher than ±1.5 were used to identify the DEGs. A total of 7133, 15, and 7120 DEGs were found in As, Fe, and As + Fe groups, respectively. Figure 4A shows the distribution of DEGs within different ranges of fold change. Most DEGs were located in the ranges of 1.5–2.5 fold and 2.5–3.5 fold. The commonality of DEGs among three groups is shown in Figure 4B, which indicates that the DEGs in As and As + Fe groups were very similar. Furthermore, qPCR was used to check the validity of microarray results. Three typical genes (*Ptgs2*, *Tgfbr1*, *Sqstm1*) related to oxidative stress, cell proliferation, and apoptotic signaling pathways were chosen. The results are shown in Table 2. Fe exposure significantly increased the fold change of these gene expressions compared with exposure to As alone, which was in accordance with the results of cDNA microarray.

In order to focus on the significantly altered biological processes, stricter criteria for DEGs of fold change > 3.0 and *p*-value < 0.001 were applied. Following these criteria, no genes were identified as DEG in the Fe group. A total of 2122 and 2121 DEGs were determined in the As and As + Fe groups, respectively. Based on the number of DEGs, exposure of As alone and combined As and Fe caused similar gene expression profiles. The biological significances of these DEGs were determined using DAVID 6.7 based on the KEGG database. Significantly changed KEGG pathways were identified on the basis of criteria that had three or more DEGs, together with a *p*-value less than 0.05. A total of 16 pathways and 20 biological processes were found in the As and As + Fe groups, respectively (Table 3), including signal transduction, nucleotide metabolism, and biosynthesis of steroids. 

### 3.5. Comparative Analysis of Toxicity in HepG2 Exposed to As Alone and Combined As and Fe

**Figure 5:**
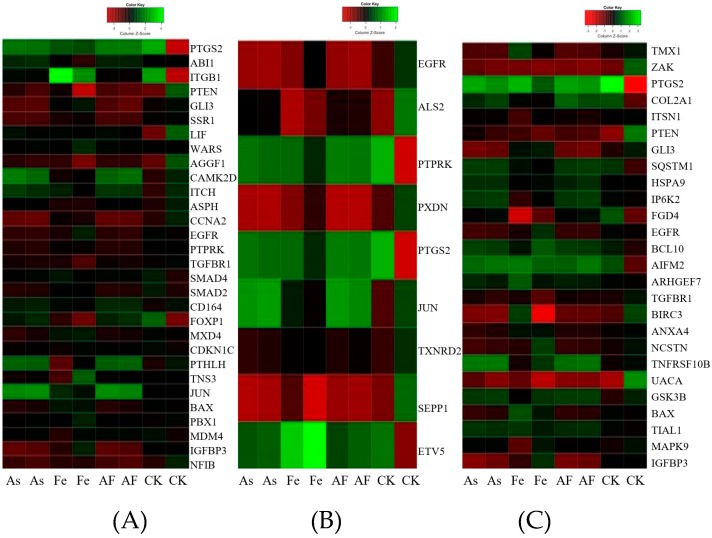
Heat map of differentially expressed genes related to altered Gene Ontology biological process based on the criteria that the ratio of fold change (As + Fe group to As group) > 1.5 and *p*-value < 0.05. (**A**) Genes related to regulation of cell proliferation; (**B**) genes related to cellular response to oxidative stress; (**C**) genes related to regulation of the apoptotic signaling pathway. CK, As, Fe, and AF mean the control, arsenic alone exposure, iron alone exposure, and co-exposure of arsenic and iron groups, respectively.

To concentrate on the influence of Fe on As toxicity, the ratio of fold change was adopted (As + Fe group to As group). A total of 429 genes were identified as DEGs with ratio > 1.5 and *p*-value < 0.05. KEGG annotation showed that the 429 DEGs were classified into nine function categories, including the Wnt signaling pathway and p53 signaling pathway, which indicates that the addition of Fe can strengthen the abnormity caused by As in signaling pathway. The GO analysis showed that these DEGs mainly participate in the regulation of cellular response to stress (GO:0033554), response to oxidative stress (GO:0006979), cell proliferation (GO:0042127), and regulation of apoptosis (GO:0042981) (Figure 5).

### 3.6. Inhibition of cell Proliferation

Plenty of studies have demonstrated that As exposure possesses the potential to influence in vitro cell proliferation by modulating several pathways, such as the MAPK-Erk, Smad, and Forkhead pathways [32,33,34,35]. Compared to the As alone group, a total of 30 DEGs in the As + Fe group were found to be involved in this process (Figure 5A). Of these DEGs, *Egfr* is a tyrosine kinase transmembrane receptor that regulates important processes in carcinogenesis, including cell proliferation. It has been suggested that As could activate ERK in PC-12 cells by binding to the cysteine-rich domains of *Egfr* [36]. Simeonova [37] found that As could induce ligand-independent *Egfr* phosphorylation and activation in UROtsa cells. Moreover, the gene *Smad2* was also found to be changed. Ten Dijke et al [38] found that the TGF-β/Smad pathway induced growth inhibitory and apoptotic responses. Sengupta [39] found that *Smad2* might have an involvement as a transcription factor for TGF-β induced cell cycle progression. These 30 DEGs in the As + Fe group indicated that the addition of Fe further increased the changes in cell proliferation caused by As exposure at transcriptomic level. Cell viability analysis provided the phenotypic results that Fe increased As toxicity on cell proliferation (Figure 1). The 30 DEGs and relevant biological pathways in the As + Fe group might be one of the reasons that Fe decreased the cell viability induced by As.

### 3.7. Oxidative Stress and Damage

Compared to exposure to As alone, the addition of Fe further increased the alteration of biological processes related to oxidative stress. A total of nine DEGs (*Als2*, *Ptprk*, *Pxdn*, *Ptgs2*, *Jun*, *Txnrd2*, *Sepp1*, *Egfr,* and *Etv5*) were involved in this process in the As + Fe group (Figure 5B). Of these DEGs, some genes need to be paid more attention for their special interaction with ROS. For example, Lee et al [40] found that homocysteine enhanced *Ptgs2* expression in murine macrophages by ROS. He et al [41] also found that chronic As exposure in human bronchial epithelial cells induced *Ptgs2* expression through HIF-1 regulation. *Txnrd2* is a mitochondrial enzyme which is critically involved in the removal of ROS and maintenance of intracellular redox balance, it is also linked to the mitochondrial respiratory chain [42]. Furthermore, at transcriptomic level, the changes to the Wnt signaling pathway and p53 signaling pathway caused by As exposure have been proven to be related to oxidative stress [43,44]. Thus, further alterations of these signal transduction pathways (As + Fe group versus As group) might be due to the increase in cellular ROS. Based on the above results, we can deduce that Fe could increase the oxidative stress and damage caused by As. The results are verified by the SOD, MDA, and DCF analysis (Figure 3). Moreover, during cDNA microarray analysis, it is very interesting that no difference was found between the As and As + Fe groups in the numbers of DEGs (Figure 4). Based on the results from the ROS assay, this might be due to the dose of As (20 μM) being so high that the influence of Fe on As toxicity could be partly covered at transcriptomic level.

### 3.8. Apoptotic singaling Pathway

Apoptosis is considered a vital component of various processes, including normal cell turnover, proper development and functioning of the immune system, hormone-dependent atrophy, embryonic development, and chemical-induced cell death [45]. A total of 26 DEGs (As + Fe group versus As group) were found to be involved in the process of regulation of the apoptotic signaling pathway (Figure 5C). Of the 26 DEGs, the gene *Sqstm1* contributes to the capacity of physiological mitochondrial function and a cell to defend itself against chemical/oxidative stress [46,47]. Auberger [48] reported that As_2_O_3_ treatment affected the expression of *Sqstm1* of CML cells, indicating the crucial role of autophagy in the effect of As_2_O_3_. In this study, we found the gene *Sqstm1* was significantly up-regulated in the As group, and the addition of Fe further increased its fold change (Table 2), indicating that Fe increased the alteration of apoptotic signaling pathways caused by As at transcriptomic level. Results of the JC1 assay further verified the result (Figure 2). Growing evidence has shown that ROS contribute to changes in the apoptotic signaling pathway by triggering the death receptor, regulating nitricoxide (NO:) and affecting mitochondria function [49,50]. Therefore, we deduced that the adverse effects of Fe on the increased As-induced apoptotic signaling pathway might be due to the increase of intracellular ROS.

## 4. Conclusions

This study showed the influence of Fe on As toxicity by in vitro assay. Our data show that Fe could decrease cell viability caused by As exposure, and increase As-induced ROS and mitochondrial depolarization. The microarray analysis further verified that Fe increased the alteration of gene expression and biological processes related to oxidative stress, cell proliferation, and apoptotic signaling pathway caused by As exposure. The increase in ROS levels under the co-exposure of As and Fe might be the important pathway where Fe increases As toxicity. In a word, an increase in endogenous Fe in the human body (or tissue) could increase the As-induced toxicity, which should be considered during the risk assessment of As.

## Figures and Tables

**Table 1:** Sequences of qPCR primers.

Gene	Forward (5’ to 3’)	Reverse (5’ to 3’)
*β-actin*	CGTACCACTGGCATCGTGAT	GTGTTGGCGTACAGGTCTTTG
*Ptgs2*	GCCCAGCACTTCACGCATCAG	GACCAGGCACCAGACCAAAGACC
*Tgfbr1*	ACATGATTCAGCCACAGATACC	GCATAGATGTCAGCACGTTTG
*Sqstm1*	AAATGGGTCCACCAGGAAACTGGA	TCAACTTCAATGCCCAGAGGGCTA

**Table 2:** Comparative analysis of expression of genes *Ptgs2*, *Tgfbr1*, and *Sqstm1* in HepG2 cells exposed to As and/or Fe for 24 h, as identified by qPCR and microarray. The fold change was normalized by control group.

Gene	Microarray		qPCR	
	As	As + Fe	As	As + Fe
*Ptgs2*	4.01	8.57	6.26	9.65
*Tgfbr1*	0.47	0.24	0.61	0.47
*Sqstm1*	2.48	4.82	10.86	20.73

**Table 3:** Altered pathways identified in As and As + Fe groups, based on the KEGG (Kyoto Encyclopedia of Genes and Genomes) database. + indicates that the pathway is significantly changed (*p*-value < 0.05).

No.	KEGG Pathway	As	As + Fe
1	Pathways in cancer		+
2	MAPK (Ras–mitogen-activated protein kinase) signaling pathway	+	+
3	Cell cycle	+	+
4	Purine metabolism	+	+
5	Pyrimidine metabolism	+	+
6	p53 signaling pathway	+	+
7	Colorectal cancer	+	+
8	DNA replication	+	+
9	ECM (Extracellular matrix)-receptor interaction		+
10	Progesterone-mediated oocyte maturation		+
11	ErbB signaling pathway		+
12	Base excision repair	+	+
13	Epithelial cell signaling in Helicobacter pylori infection	+	+
14	Aminoacyl-tRNA biosynthesis	+	+
15	Lysine degradation	+	+
16	Pyruvate metabolism	+	+
17	Bladder cancer	+	
18	Notch signaling pathway		+
19	Alanine, aspartate and glutamate metabolism	+	+
20	Porphyrin and chlorophyll metabolism	+	
21	One carbon pool by folate		+
22	Steroid biosynthesis	+	+
